# Body temperature instability and respiratory morbidity in the very low birth weight infant: a multiple case, intensive longitudinal study

**DOI:** 10.1186/s12887-020-02351-y

**Published:** 2020-10-20

**Authors:** Jane L. Ralphe, Susan G. Silva, Robin B. Dail, Debra H. Brandon

**Affiliations:** 1 University of Wisconsin-Madison School of Nursing, University of Wisconsin, 701 Highland Ave. Madison, WI WI 53705 Madison, USA; 2 Duke University School of Nursing, Duke University, NC Durham, USA; 3 Duke University School of Medicine, Duke University, NC Durham, USA; 4 University of South Carolina College of Nursing, University of South Carolina, SC Columbia, USA

**Keywords:** Premature infant, Very low birth weight infant, Chronic lung disease, Respiratory morbidity, Bronchopulmonary dysplasia, Desaturations, Bradycardia with desaturations, Respiratory support

## Abstract

**Background:**

Very low birth weight (VLBW) infant thermal instability upon neonatal intensive care unit admission has been associated with respiratory morbidity; however, the association between ongoing thermal instability and respiratory morbidity remains unclear.

**Methods:**

A longitudinal data analysis was conducted on 12 VLBW infants. Chronic respiratory morbidity risk was defined as supplemental oxygen requirement (FiO_2_) or scheduled diuretic dosing at 36 weeks post-menstrual age. Acute respiratory morbidity was quantified as desaturations (SpO_2_<90%), bradycardia with desaturations (HR<100 and SpO_2_<90%), apnea, increase in FiO_2_ requirement, or increase in respiratory support. Multi-level, mixed-effects models and regression analysis examined the relationships between body temperature over the first 14 days of life and respiratory morbidities.

**Results:**

Body temperature was not associated with chronic respiratory morbidity risk (*p=*0.2765). Desaturations, bradycardia with desaturations, increased FiO_2_ requirement, and increased respiratory support were associated with decreased body temperature (*p<*0.05). Apnea was associated with increased body temperature (*p*<0.05). The covariate-adjusted risk of desaturations (*aOR*=1.3), bradycardia with desaturations (*aOR*=2.2), increase in FiO_2_ requirement *(aOR*=1.2), and increase in respiratory support (*aOR=*1.2) were significantly greater during episodes of hypothermia.

**Conclusion:**

VLBW infants are dependent on a neutral thermal environment for optimal growth and development. Therefore, the significant associations between hypothermia and symptoms of acute respiratory morbidity require further study to delineate if these are causal relationships that could be attenuated with clinical practice changes, or if these are concurrent symptoms that cluster during episodes of physiological instability.

## Background

Approximately 50,000 very low birth weight (VLBW) infants (< 1500 g) are born in the United States each year [1]. The most common complication of very premature birth is bronchopulmonary dysplasia (BPD) [2, 3], which occurs in approximately 50% of these infants [4, 5]. BPD occurs due to the arrest of normal lung development following premature birth. It is characterized by large, sparse alveoli and dysmorphic pulmonary vasculature, resulting in a decrease in surface area available for gas exchange [3, 6]. Although advances in neonatal care have reduced the rate of severe BPD and its associated mortality, the incidence of BPD has risen due to the improved survival of extremely premature infants (< 1000 grams) [5, 7]. Many risk factors have been associated with the development of BPD; however, only a portion of infants with these shared risk factors develop this morbidity, thus complicating the prevention and treatment of disease [4].

Hypothermia and hyperthermia, in the early hours of life, have been associated with the development of BPD in VLBW infants [8–10]. While the ideal body temperature for these infants is unclear, temperatures < 36.5^o^ C and > 37.2^o^ C have been associated with an increased risk of BPD [10]. Due to extreme thermoregulatory system immaturity, the VLBW infant is unable to maintain euthermic body temperatures independently [11–13]. Both autonomic dysfunction [14–16] and skin immaturity [13] contribute to thermal instability in these infants. Typically, neonates are reliant on nonshivering thermogenesis (NST) (i.e., heat production via sympathetic stimulation of brown adipose tissue ) during periods of hypothermia [12, 17]; however, NST is ineffective in VLBW infants because elements essential to brown fat metabolism (e.g., thermogenin and 5’-monodeiodinase) are limited, thus oxygen is metabolized for heat production [12, 18, 19]. However, due to the extreme immaturity of the pulmonary system, VLBW infants are unable to compensate for an increase in oxygen consumption, therefore if hypothermia persists, hypoxemia and acidosis can ensue [12]. This hypoxemia can manifest as desaturations (SpO_2_ < 90%) or bradycardia (HR < 100) with desaturations (B/D) [20]. Recent studies have found an association between desaturations and long-term morbidity and mortality in very premature infants, including chronic lung disease [21–23]. Supplemental oxygen (FiO_2_ > 21%) and increased respiratory support (RS) (e.g., positive inspiratory pressure (PIP), positive end-expiratory pressure (PEEP)) are used to treat hypoxemia and respiratory acidosis, respectively. Reactive oxygen species (ROS), a product of supplemental FiO_2_, and positive pressure from RS can result in pulmonary epithelial damage, which can stimulate and/or sustain the inflammatory process that is strongly associated with the development of BPD [6, 24, 25].

While NICU admission temperature instability has been implicated in the development of BPD in VLBW infants [8–10], only a portion of infants with admission temperature instability develop this morbidity. In addition, recent studies have been unable to validate this relationship, thus adding confusion to the influence of this potential risk factor [26, 27]. Although VLBW infants are susceptible to thermal instability for weeks following NICU admission, few studies have examined the impact of longitudinal body temperature instability on acute or chronic respiratory morbidity. Therefore, the purpose of this study was to examine the relationship between VLBW infant body temperature instability over the first 14 days of life and symptoms of acute and chronic respiratory morbidity. This study specifically examined the association between VLBW infant body temperatures and both chronic respiratory morbidity risk (CRMR) (i.e., FiO_2_ requirement or scheduled diuretic use at 36 weeks postmenstrual age (PMA) and biomarkers for acute respiratory morbidity (i.e., desaturations, B/D, apnea, increased FiO_2_ requirement, and increase in RS).

## Methods

### Subjects

This was a secondary analysis of data from 12 very premature infants enrolled in the parent study, “*Body Temperature and Vasomotor Tone in Preterm Infants*,”(NIH/NINR: 1R15NR012157-0; RWJF: 68041), an intensive exploratory study conducted at a North Carolina university hospital from 2010–2013 that examined vasomotor tone maturation and associated morbidity and mortality. Eligibility criteria for the parent study were infants < 29 weeks gestational age (GA) at birth and a birthweight (BW) of < 1200 grams, and it included 30 infants: 22 infants completing all data collection [28]. This secondary analysis included 12 infants from the parent study. Infants transferred before 34 weeks postmenstrual age (PMA) (two weeks prior to BPD diagnoses) (N = 5) or discharged before 36 weeks PMA (N = 1) were excluded from this secondary analysis. An additional infant (N = 1) was excluded because some data were no longer accessible for analysis. Because birthweight is a significant risk factor for BPD and thermal instability [5, 16], a sample that equally represented three birthweight categories (< 800 grams, 800–900 grams, > 900 grams) was then chosen from the remaining infants (N = 15). This sample (N = 12) included four infants per birthweight category. The parent study and this secondary analysis was approved by Duke Health Institutional Review Board.

### Measurements

Infants were stabilized under radiant heat, then transferred to a pre-warmed Draeger Caleo incubator within 4–6 hours of life (Knobel-Dail et al., 2017). Per study site standard of care, infants were placed on servo-control mode with a setting of 36.5 °C to 37.2 °C, with a relative humidity setting based on birthweight (≤ 750 grams = 75–80%; >750 grams = 0-40%). Using a skin temperature probe, servo-control mode continuously monitors skin temperature, adjusting heat output to achieve or maintain the servo temperature setting. The servo temperature setting (servo set point) was retrieved from the electronic health record (EHR). Minute to minute abdominal temperatures were measured by covered Y series Steri-Probe® skin temperature probes, which were covered with a reflective cover (Model 499B, Cincinnati Sub-Zero, Cincinnati, OH) [28]. Nurses repositioned infants and examined skin at probe sites throughout each day according to standard care [28]. Temperature probe positions were changed a minimum of every 6–8 hours, with the infant’s skin condition recorded on the infant’s case study log [28]. With secure and appropriated probe placement, the skin temperature of VLBW infants is an adequate proxy for their body temperature [29, 30]. The thermistor was attached to a 4-channel data logger, model SP-1400-44Y (Veriteq Instruments; Vaisala; Richmond, British Columbia, CA), which sampled and stored temperatures every minute for 2 weeks. Body temperatures below 33.0^o^ C and above 39.0^o^ C were removed during parent study data cleaning, as they were thought to represent errors in data collection (i.e., insecure probe placement or probe removal for repositioning) [28]. These temperatures were marked as missing and were excluded from analysis. Heart rate (HR) was monitored continuously with the infant’s General Electric Healthcare cardiopulmonary monitor. Oxygen saturations (SpO_2_) were collected using Masimo Radical-7 pulse oximeters (Masimo Corporation, Irvine, CA) with 10-second averaging. Missing SpO_2_ measurements or measurements equal to zero were excluded from analysis. All minute-to-minute measurements (~ 20,000 measures per data type) were downloaded into a laptop computer. Additional respiratory data, including apnea episodes, FiO_2_ requirement, RS settings (i.e., PIP, PEEP, respiratory rate (RR)), surfactant and diuretic dosing, and FiO_2_ requirement at 28 days and 36 weeks PMA were extracted from EHR review. Infant demographics and additional risk factors for acute respiratory morbidity and CLD were also extracted from the EHR, including: GA, BW, sex, maternal diagnosis of chorioamnionitis, Apgar scores at 1 and 5 minutes, infant infection (i.e., sepsis or presumed sepsis), small for gestational age (SGA), intrauterine growth restriction (IUGR), and diagnosis of patent ductus arteriosus (PDA). See Table 1 for analysis variables and their definitions.

**Table 1:** Analysis variables

Variable	Definition
Temperature	
Body	Abdominal skin temp in ^o^C
Hypothermia	Body temp < 36.5^o^C
Hyperthermia	Body temp > 37.2^o^C
Euthermia	Body temp 36.5^o^C to 37.2^o^C
Hypothermic episode	Begins when body temp < 36.5^o^C & ends when body temp ≥ 36.5^o^C (in min)
Hyperthermic episode	Begins when body temp < 37.2^o^C & ends when body temp ≥ 37.2^o^C (in min)
Acute Respiratory Morbidity	
Desaturation	SpO_2_ < 90%
Brady/Desaturation	Heart rate < 100 & SpO_2_ < 90%
Apnea	Time of apnea plus 15 min prior & 5 min after
FiO_2_ increase	Time of FiO_2_ increase plus 15 min prior to & 5 min after
RS increase	Time of PIP, PEEP, or RR increase plus 15 min prior to & 5 min after
Chronic Respiratory Morbidity	
CRMR	Diagnosis of BPD or SD
BPD	FiO_2_ > 21% at 28 DOL or 36 weeks PMA
SD	No diagnosis of BPD, but receiving scheduled diuretic dosing for respiratory symptoms at 36 weeks PMA
Covariates	
Chorioamnionitis	Maternal diagnosis of chorioamnionitis
GA	Gestational age at birth in weeks
Birthweight	Birthweight in grams
SGA	Birthweight < 10th percentile on growth curve
Sex	Female or Male
Apgar Scores	Apgar score at 1 & 5 minutes
Surfactant	Received at least one dose of surfactant
Infection	Sepsis or presumed sepsis during the first 14 DOL

*temp* temperature; *FiO*_*2*_ fraction of inspired air; *SpO*_*2*_ peripheral capillary oxygen saturation; *min* minute; *RS* respiratory support; *BPD* bronchopulmonary dysplasia; *SD* scheduled diuretics dosing; *CRMR *chronic respirtory morbidity risk; *DOL* days of life; *PMA* postmenstrual age; *GA* gestational age; *SGA* small for gestational age; *IUGR* intrauterine growth restriction

Abdominal temperature, HR, SpO_2_, and EHR data for each infant were concatenated and archived in a SAS case dataset (one dataset per infant) using ^®^SAS statistical software version 9.4 (Cary, NC). The 12 infant datasets were then merged into a final analysis dataset. Analysis variables in this final dataset included measures of body temperature, biomarkers for acute respiratory morbidity, chronic respiratory morbidity risk, and covariates. The covariates SGA and IUGR were combined into a single covariate during analysis as infants with IUGR in this study also met criteria for SGA (i.e., BW < 10% on growth curve).

### Data analysis

Descriptive statistics were used to summarize infant characteristics and analysis variables during the first 14 days of life (DOL). Non-directional statistical tests were performed with the level of significance set at 0.05 for each test. The significance level was not adjusted for the multiple outcomes and tests for this exploratory analysis of 12 VLBW infants. Effect sizes and their 95% confidence intervals (CIs) were calculated to address clinical relevance.

Hierarchical multi-level, mixed-effects models for intensive longitudinal data were used to examine the association between the infant body temperatures during the first 14 DOL and both CRMR and biomarkers for acute respiratory morbidity. Random coefficients regression models (RRMs), a type of multi-level, mixed-effects model for intensive longitudinal data, were used for continuous outcomes, while Generalized Linear Mixed Models (GLIMMIX) were applied for binary outcomes. A multi-level approach was applied because level-3 of such models allowed us to adjust for nesting within each infant. The multi-level models also included six infant covariates: (1) infant GA, (2) BW, (3) Apgar score at 5 minutes, (4) infection, (5) sex, and (6) surfactant dosing. SGA/IUGR was omitted as a covariate due to its collinearity with GA (Spearman r_s_ = 0.66). Similarly, Apgar score at one minute (Spearman r_s_ = -0.68), chorioamnionitis (Spearman r_s_ = 0.55), and PDA (Spearman r_s_ = -0.52) were excluded due to collinearity with surfactant. The fixed effects in the multi-level modes were the measured outcomes (i.e., CRMR, desaturations, B/D, apnea, increase in FiO2 requirement, or increase in RS) and the six infant covariates, while the random effect was the infant.

## Results

Infant characteristics and clinical variables are found in Table 2. The median GA was 27.1 weeks, and the median BW was 865 grams. Infants were 58% female and 67% non-Hispanic Black. The remaining infants were non-Hispanic White (25%) and Hispanic (8%).

**Table 2:** Demographic and clinical variables

Respiratory Outcome		Respiratory Support Received	GA	BW	Sex	Race	Chorio	Surf	Infection
CRMR	BPD	MV, CPAP	25.9	880	M	B	No	Yes	Yes
	BPD	MV, CPAP	27.1	820	F	B	No	Yes	Yes
	SD	MV, CPAP	26.1	660	F	H	No	Yes	Yes
	SD	MV, CPAP	26.1	850	M	B	No	Yes	No
	SD	MV, CPAP	26.3	760	M	B	Yes	No	Yes
	SD	CPAP, HFNC, RA	27.4	1040	M	B	Yes	No	Yes
	SD	MV, CPAP, HFNC	27.6	730	M	W	No	Yes	Yes
	SD	MV, CPAP	27.9	740	F	B	No	Yes	Yes
No CRMR		MV, CPAP	27.1	880	F	B	Yes	Yes	Yes
		MV, CPAP	27.1	940	F	W	No	Yes	No
		MV, CPAP, RA	27.1	1040	F	W	No	Yes	No
		CPAP, HFNC, RA	27.6	1050	F	B	No	No	No

### Body temperature trajectory over the first 14 days of life

RRMs were used to determine and describe the trajectory of infant body temperatures across the 14 days. The fixed effects were DOL and the six covariates, while random effects were infant and the infant-by-DOL interaction trajectories. Outcome was body temperature across the 14 days, which did not significantly change across days (*p =* 0.526), and none of the covariates were significantly associated infant body temperature (all *p >* 0.05). Since there was not a significant change in body temperature across days, DOL was not included as a predictor in the multi-level model analysis models.

### Body temperatures and chronic respiratory morbidity risk

Descriptive statistics were used to examine the association between episodes of hypothermia and hyperthermia with the outcome of CRMR. Minute-by-minute data with missing temperatures were excluded from the analysis. Hypothermic and hyperthermic episodes were analyzed by infant case, then merged for group analysis (*N* = 12). Following removal of missing abdominal temperatures, an average of 22.3 hours (20.3–23.1 hours) per infant per day were assessed over the 14 days. As a group (*N* = 12), infants spent almost one-third of the assessed time hypothermic (Median = 30.5%, IQR = 26.7% − 49.1%), and the median duration of each hypothermic episode was 26.8 minutes (IQR = 20.2–38.7). Infants without CRMR (N = 04) spent more time hypothermic (Median = 51.8%, IQR = 35.7–63.6%) than infants with CRMR (*N* = 08) (Median = 27.6%, IQR = 26.7–34.2%), and the duration of hypothermic events were longer in infants without CRMR (Median = 42.4 min, IQR = 26.1–62.7 min) than infants with CRMR (Median = 22.4 min, IQR = 20.2–34.4 min). The occurrence of hyperthermia was similar among infants with and without CRMR (CRMR: Median = 6.3%, IQR = 4.4% − 10.5%; without CRMR: Median = 6.5%, IQR 3.4–9.5%), yet the duration of hyperthermic episodes was shorter among infants with than without CRMR (CRMR: Median = 36.3 minutes, IQR = 30.6–55.7; without CRMR: Median = 47.2 min, IQR = 39.1–50.9).

Multi-level models were then used to examine the association between infant body temperatures and CRMR. Infant body temperatures were not significantly associated with the outcome of CRMR (*p =* 0.2765). However, the adjusted mean body temperature of infants with CRMR was euthermic, while the adjusted mean body temperature of the infants without CRMR were hypothermic. Very small effect sizes for these statistically significant relationships were observed (both Cohen’s *d* < ± 0.20). All covariates were non-significant (all *p* > 0.05). Table 3 presents the adjusted means and details of RRM.

**Table 3:** Relationships between infant body temperatures and chronic respiratory morbidity risk, oxygen desaturations, and bradycardia with oxygen desaturations

Outcome	F *p*-value	Adjusted Mean ± SE^a^	Cohen *d*^b^	Cohen *d* 95% CI^b^
CRMR	0.2765		0.01	0.00, 0.02
Yes		36.69 ± 0.14		
No		36.23 ± 0.25		
O_2_ desat	< 0.0001		-0.01	-0.02, 0.01
Yes		36.49 ± 0.05		
No		36.59 ± 0.05		
B/D	< 0.0001		0.03	0.02, 0.04
Yes		36.29 ± 0.06		
No		36.58 ± 0.06		

*CRMR* chronic respiratory morbidity risk, *O*_*2*_ *desat* oxygen desaturation, *B/D* bradycardia with oxygen desaturation ^a^Mean and SE adjusted for fixed, random, and nesting effects ^b^Cohen d effect sizes: small= ±0.20, medium= ±0.50, large = ±0.80

### Body temperatures and desaturations and bradycardia with desaturations

Multi-level models were also used to examine the association between infant body temperatures and desaturations and B/D. Desaturations and B/Ds were significantly associated with lower body temperatures (both *p* < 0.0001). Very small effect sizes for these statistically significant relationships were observed (both *d* < ± 0.20). All covariates were non-significant (all *p* > 0.05). Table 3 presents the adjusted means and details of RRM.

### Body temperatures and episodes of apnea, increases in FiO2 and RS requirement

Bivariate regression and GLIMMIX were used to examine the association between infant body temperatures and documented episodes of apnea, increases in FiO_2_ requirement, and increases in RS. Each episode included the recorded respiratory event (e.g. apnea), as well as the 15 minutes preceding and the five minutes following each event. These episodes were created to assess the relationship between the respiratory event and it’s preceding temperatures.

Table 4 shows the results of regression analysis of apnea, increase in FiO2 requirement, increase in RS, and associated covariates in relation to infant body temperature. Apnea was significantly associated with warmer body temperatures (*p* = 0.0318*)*, and all covariates were significant. Increase in FiO_2_ requirement (*p* = 0.0298) and increase in RS (*p <* 0.0001) were significantly associated with decreased body temperatures. All covariates were significantly associated with all infant body temperature (all *p* < 0.0001), with larger BW, younger GA, higher Apgar scores, male sex, and surfactant dosing associated with higher body temperatures, while presence of infection was associated with decreased body temperatures.

**Table 4:** Relationship between body temperature and apnea, increases in oxygen requirement, and increases in respiratory support

	^*a*^*b*	SE	*B*	*t*	^b^Adj *R*^*2*^
Body Temperature					0.043
^ *^Apnea	0.025	0.012	0.005	2.15	
^ **^Birthweight	0.000	0.000	0.021	8.26	
^ **^Sex	0.249	0.004	0.172	64.68	
^ **^GA	-0.067	0.003	-0.059	-25.03	
^ **^Apgar	0.084	0.001	0.147	65.29	
^ **^Surfactant	0.082	0.005	0.050	17.92	
^ **^Infection	-0.071	0.004	-0.046	-17.11	
Body Temperature					0.043
^ *^Increase in FiO_2_	-0.014	0.013	-0.002	-1.04	
^ **^Birthweight	0.001	0.000	0.017	6.99	
^ **^Sex	0.247	0.004	0.171	64.54	
^ **^GA	-0.070	0.003	-0.062	-26.20	
^ **^Apgar 5	0.084	0.001	0.148	65.75	
^ **^Surfactant	0.088	0.005	0.054	19.10	
^ **^Infection	-0.073	0.004	-0.048	-17.65	
Body Temperature					0.042
^ **^Increase in RS	-0.077	0.020	-0.008	-3.81	
^ **^Birthweight	0.000	0.000	0.019	7.51	
^ **^Sex	0.246	0.004	0.170	63.95	
^ **^GA	-0.068	0.003	-0.060	-25.35	
^ **^Apgar	0.082	0.001	0.145	63.97	
^ **^Surfactant	0.087	0.005	0.054	18.97	
^ **^Infection	-0.074	0.004	-0.049	-17.80	

*GA* gestational age, *FiO2* supplemental oxygen, *RS* respiratory support

^a^*b* = unstandardized trajectory slope

^b^*Adj R*^*2*^ = R-squared for the model adjusted for number of explanatory variables

^*^p < 0.05; ^**^p < 0.0001

### Hypothermia and hyperthermia and chronic and acute respiratory morbidity

**Fig. 1:**
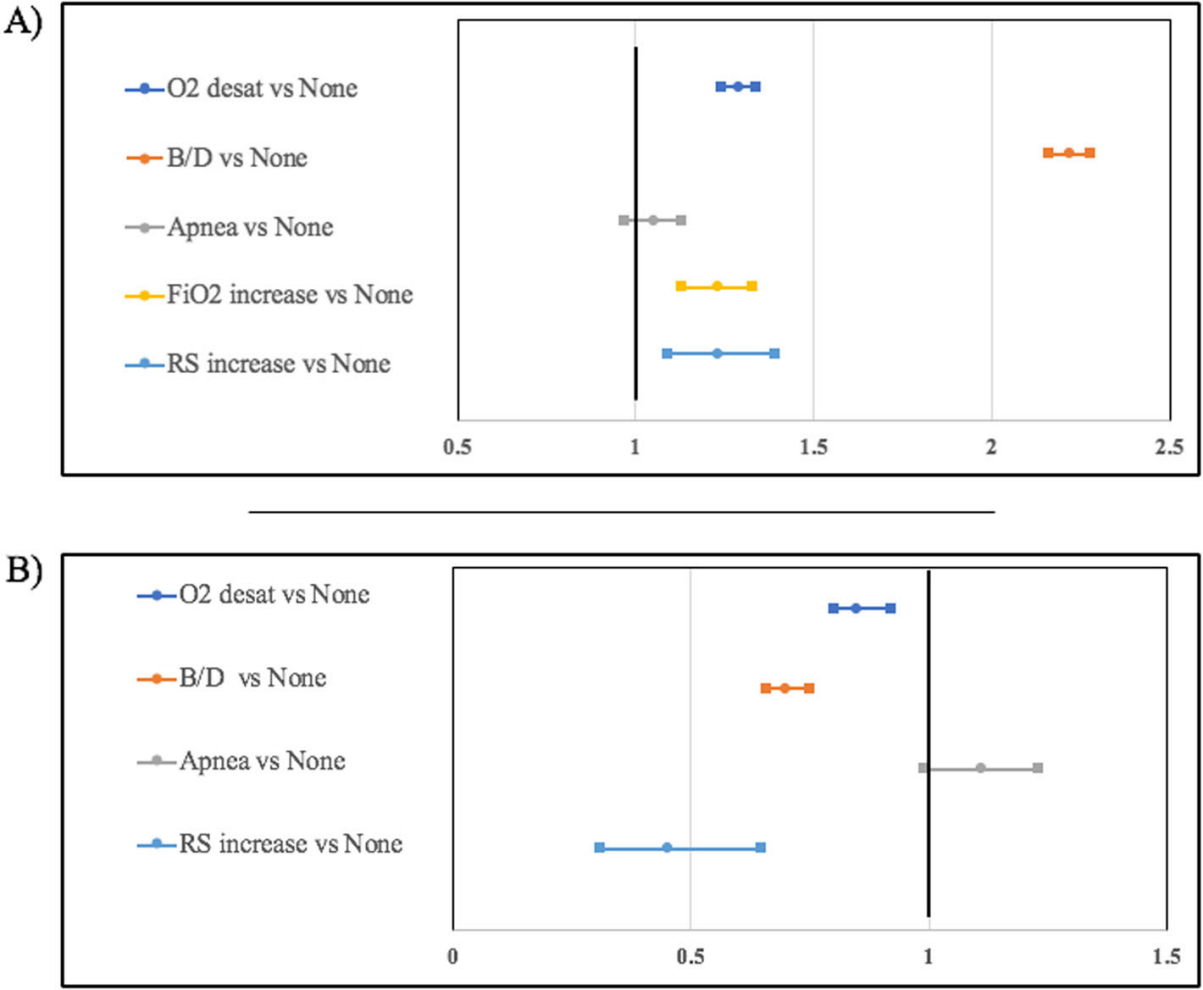
Adjusted odds ratios and 95% confidence intervals for symptoms of acute respiratory morbidity during episodes of hypothermia (<36.5^o^C) (**a**) and hyperthermia (>37.3^o^C) (**b**)

Desaturations, B/D, increases in FiO_2_ requirement, and increases in RS were significantly associated with hypothermia and hyperthermia (all *p* < 0.0001). The adjusted odds of hypothermia was 1.3 times higher during desaturations, 2.2 times higher during B/D, and 1.2 times higher during increases in FiO2 requirement and increases in RS. The adjusted odds of hyperthermia was 1.2 times lower during desaturations, 1.4 times lower during B/D, and 2.2 times lower during increases in RS. See Fig. 1 for a summary of the aORs. The episodes of increased FiO_2_ requirement during hyperthermia were too few to analyze. Covariates were associated with hypothermia, including increased GA during B/D (*p* = 0.0057), lower Apgar scores during increases in FiO2 requirement (*p* = 0.0220), and smaller BW during increases in RS (*p* = 0.0390). Covariates associated with hyperthermia included lower Apgar score during desaturation (*p* = 0.0170), increased Apgar score during B/D (*p* = 0.0170), and smaller BW during increases in RS (*p* = 0.0051). The remaining covariates were not significantly related to hypothermia or hyperthermia (all *p* > 0.05).

## Discussion

We examined the associations between infant body temperatures and CRMR, as well as symptoms of acute respiratory morbidity in the first 14 days after birth in 12 VLBW infants. Our major findings were that decreased body temperatures were associated with desaturations, B/D, increases in FiO2 requirement, and increases in RS. We also found that there was an increased risk of these events occurring during episodes of hypothermia, and a decreased risk during episodes of hyperthermia.

The adjusted mean infant body temperature was 36.5^o^C each day of the first 14 DOL. This borderline euthermia occurred despite higher adjusted mean servo set point temperatures (36.7-36.8^o^C). These infants also spent almost one-third of the 14 days hypothermic, with each hypothermic episode lasting almost 27 minutes. Given the intensive care necessary for these highly vulnerable infants, these frequent hypothermic episodes, along with body temperatures consistently below servo set point temperatures, likely reflect frequent disruption of the neutral thermal environment (i.e., heat and humidity loss) by the opening of the incubator portholes to provide essential care, as well as contact heat loss through caregiver hand contact [13, 17]. In addition, the high frequency and extended duration of hypothermic episodes demonstrate an increased risk of hypothermia associated morbidity (e.g., BPD, NEC, retinopathy of prematurity, infection) among these infants [10].

While only two infants met the NIH criteria for BPD, an additional six infants were receiving diuretics for respiratory symptoms at 36 weeks PMA. Historically, BPD has been diagnosed as oxygen dependency at ≥ 36 weeks postmenstrual age (PMA) [31, 32]. However, this clinical definition has been refined by the NIH to include very premature infants with an oxygen requirement at 28 days of life (DOL), as well as a severity assignment (mild, moderate, or severe) based on oxygen requirement at 36 weeks PMA [32]. The definition of BPD continues to be a topic of debate since it manifests differently across infants [32, 33], and because this diagnosis does not capture all infants with chronic pulmonary disease related to their premature birth [34, 35]. The use of diuretics also remains controversial, yet studies have shown that it remains one of the most utilized medications in the NICU, with over 50% of infants receiving dosing during hospitalization [36, 37]. Chronic pulmonary insufficiency of prematurity is a global concept that includes chronic respiratory morbidities diagnosed during the infant’s neonatal intensive care unit (NICU) hospitalization through early childhood (e.g., reactive airway disease) [33]. Because infants without a diagnosis of BPD have been shown to have CLD (e.g., chronic pulmonary insufficiency) later in life [34, 35], we considered CRMR to be a broad concept that includes, but is not limited to BPD. Therefore, we combined BPD and diuretic use into a more general CRMR category to capture infants receiving prescribed treatment (i.e., supplemental FiO_2_, scheduled diuretics) for residual lung disease symptoms at 36 weeks PMA.

The four infants without CRMR (i.e., BPD or scheduled diuretics) in this study were all female. In addition, seven infants (88%) in the CRMR group were treated for infection during the first 14 days of life, while only one infant (25%) without CRMR was treated for infection. Although this is a very small sample, these results are consistent with previous findings that link male sex [24] and infection [38] with VLBW infant respiratory morbidity.

Infants without CRMR had lower body temperatures than those with CRMR. Although this association was not statistically significant, the adjusted mean body temperature of those without CRMR was hypothermic, while the adjusted mean body temperature of those with CRMR was euthermic. Infants without CRMR also spent more time hypothermic, and the episodes of hypothermia were longer than infants with CRMR. While these findings contradict previous studies linking admission hypothermia and chronic respiratory morbidity [10], this study assessed infant body temperatures beyond admission. In addition, these results are consistent with BPD animal model studies that found hypothermia to be protective against inflammatory mechanisms associated with the disease [39, 40]. When we examined individual infant temperatures over time, the infant with the lowest median body temperature (Median = 35.8^o^C, IQR = 35.8–36.6^o^C) across the 14 days did not have CRMR. Yet, this infant developed necrotizing enterocolitis (NEC): a morbidity also associated with hypothermia [10]. Given the exploratory nature of this study, an association between hypothermia beyond admission and decreased CRMR risk cannot be concluded. However, if hypothermia is protective against CRMR, establishing a VLBW infant body temperature range that minimizes CRMR over time, without increasing the risk of mortality and other morbidities (e.g., NEC) should become a priority.

Consistent with previous research, this analysis showed an association between apnea and rising infant body temperatures [41, 42]. These findings further demonstrate the significance of body temperature extremes and the importance of maintaining infant thermal stability. Due to the low number of apnea events occurring during episodes of hyperthermia, we were unable to assess this association.

Hypothermia has also been associated with respiratory depression, which can result in desaturations, B/D and the need for increased RS [13, 20]; however, to our knowledge, this is the first study to assess and find an association between decreased VLBW infant body temperatures over time and both desaturations and B/D. While the effect sizes for these associations were very small, the odds of experiencing an desaturations or B/D were significantly higher during hypothermia and significantly lower during hyperthermia. However, it is important to delineate the meaning behind these associations, as it is unclear if desaturations and B/D are biomarkers for acute respiratory morbidity associated with hypothermia, or if hypothermia is a biomarker for physiological instability that cluster with these other symptoms of instability.

Consistent with previous research, this study found a significant association between increasing RS and infant body temperature instability [43]. Lower body temperatures were associated with an increase in both FiO_2_ requirement and respiratory support, and the odds of hypothermia associated with those changes was significantly higher as well. Several infant characteristics contributed to the prediction of increases in FiO_2_ requirement and respiratory support; BW was associated with lower body temperatures, while male sex and higher Apgar score were associated with higher body temperatures. This association between male sex and increased body temperature is an important finding given that male infants have an increased risk of CLD [44, 45], and this analysis also found an association between increased body temperatures and CRMR.

Analysis from this study also showed an inverse relationship between GA and body temperature. Per the study site standard of care, infants ≤ 750 grams were placed in 75–80% humidity, while infants > 750 grams were placed in 40% humidity during the first 14 days of life. Given smaller infants in this study had warmer body temperatures over time, these results support previous findings that environmental humidity protects very premature infants from heat loss via insensible water loss [46].

There are limitations to this study. This was an intensive exploratory study; thus, the sample size was small. Also, the sample was somewhat homogenous, lacking equal ethnic and racial representation. Given the physiologic data were previously collected, data analysis was limited by the parent study design. Also, the sample represented a single NICU; therefore, the generalizability to other VLBW infants may be limited, as the infant temperatures and respiratory outcomes may be associated with the standard of care and institutional guidelines.

## Conclusion

This study found significant associations between adjusted mean temperatures and symptoms of acute respiratory morbidity; however, to examine causal relationships, larger longitudinal studies are needed. These studies should examine the temporal relationships between infant body temperature instability and desaturations, B/D, increased FiO_2_ requirement, and increased respiratory support. Advanced longitudinal analysis can assist in delineating if thermal instability is the causative factor of these symptoms or if thermal instability is a biomarker of physiological instability that manifests concurrently with these respiratory symptoms. Also, these types of studies are needed to adequately explore the relationship between infant body temperature instability over time and CRMR. Finally, the ideal temperature range that minimizes morbidity risk as VLBW infants grow and develop over time should be determined. If a true causal relationship between longitudinal temperature instability and CRMR is found and an ideal body temperature range that reduces infant risk is delineated, intervention studies can be developed, and clinical guidelines can be adapted to reduce infant risk.

## Data Availability

The datasets used and/or analyzed during the current study are available from the corresponding author on reasonable request.

## Abbreviations

VLBW: Very low birthweight
BW: Birthweight
BPD: Bronchopulmonary dysplasia
CRMR: Chronic respiratory morbidity risk
SD: Scheduled diuretics
NST: Nonshivering thermogenesis
temp: Temperature
surf: Surfactant
chorio: Chorioamnionitis
FiO_2_: Fraction of inspired oxygen
SpO_2_: Oxygen saturation
HR: Heart rate
B/D: Bradycardia with desaturation
RS: Respiratory support
RR: Respiratory rate
PIP: Positive inspiratory pressure
PEEP: Positive end expiratory pressure
ROS: Reactive oxygen species
GA: Gestational age
SGA: Small for gestational age
IUGR: Intrauterine growth restriction
PDA: Patent ductus arteriosus
CI: Confidence intervals
RRM: Random coefficient regression models
GLIMMIX: Generalized linear mixed models
